# Improving the Physicochemical and Biopharmaceutical Properties of Active Pharmaceutical Ingredients Derived from Traditional Chinese Medicine through Cocrystal Engineering

**DOI:** 10.3390/pharmaceutics13122160

**Published:** 2021-12-15

**Authors:** Danyingzi Guan, Bianfei Xuan, Chengguang Wang, Ruitao Long, Yaqin Jiang, Lina Mao, Jinbing Kang, Ziwen Wang, Shing Fung Chow, Qun Zhou

**Affiliations:** 1 Hubei Key Laboratory of Natural Medicinal Chemistry and Resource Evaluation, School of Pharmacy, Tongji Medical College, Huazhong University of Science and Technology, Wuhan 430030, China; guandanyingzi@hust.edu.cn (D.G.); ruitao.long@gmail.com (R.L.); M202075574@hust.edu.cn (Y.J.); m202175560@hust.edu.cn (L.M.); m202178120@hust.edu.cn (J.K.); wangziwen2020@163.com (Z.W.); 2 Department of Pharmacology and Pharmacy, Li Ka Shing Faculty of Medicine, The University of Hong Kong, Hong Kong, China; bxuan@connect.hku.hk; 3 Pharmaceutical Materials Science and Engineering Laboratory, Department of Pharmaceutics, College of Pharmacy, University of Minnesota, Minneapolis, MN 55455, USA; wang4889@umn.enu

**Keywords:** pharmaceutical cocrystal, physicochemical properties, active pharmaceutical ingredients, solubility, dissolution, stability, traditional Chinese medicine, cocrystal engineering

## Abstract

Active pharmaceutical ingredients (APIs) extracted and isolated from traditional Chinese medicines (TCMs) are of interest for drug development due to their wide range of biological activities. However, the overwhelming majority of APIs in TCMs (T-APIs), including flavonoids, terpenoids, alkaloids and phenolic acids, are limited by their poor physicochemical and biopharmaceutical properties, such as solubility, dissolution performance, stability and tabletability for drug development. Cocrystallization of these T-APIs with coformers offers unique advantages to modulate physicochemical properties of these drugs without compromising the therapeutic benefits by non-covalent interactions. This review provides a comprehensive overview of current challenges, applications, and future directions of T-API cocrystals, including cocrystal designs, preparation methods, modifications and corresponding mechanisms of physicochemical and biopharmaceutical properties. Moreover, a variety of studies are presented to elucidate the relationship between the crystal structures of cocrystals and their resulting properties, along with the underlying mechanism for such changes. It is believed that a comprehensive understanding of cocrystal engineering could contribute to the development of more bioactive natural compounds into new drugs.

## 1. Introduction

In China, there are several thousand natural medicines available in nature for preventing and curing diseases [1]. Herbal medicines utilized under the guidance of Chinese medical theory established in ancient China are collectively termed as traditional Chinese medicines (TCMs). A large number of compounds have been extracted and isolated from TCMs, mainly being grouped as flavonoids, coumarins, alkaloids, glycosides, terpenoids, phenolic acids and lignans, etc. For example, there are at least 341 compounds extracted from the genus *Lindera* plants, including sesquiterpenoids, alkaloids, flavonoids, butanolides, lucidones and phenylpropanoids [2]. The majority of active pharmaceutical ingredients (APIs) of TCMs (T-APIs) exist in crystalline solids. Many of them possess a wide range of pharmacological activities, such as anti-oxidation, anti-inflammatory, anti-viral, anti-bacteria, anti-tumor and anti-malaria properties, enhancing immunity, regulating blood lipids and lowering blood sugar [3,4,5]. These T-APIs have gained increasing attention for their therapeutic effects on a variety of diseases, including cardiovascular disease, diabetes mellitus, Alzheimer’s disease, hepatic cirrhosis and cancers [3]. Thus, they are worthy of further evaluation for their underlying mechanisms in disease prevention or therapy. For example, paclitaxel, a drug derived from the bark of the Pacific yew tree, is currently used for the first-line treatment of ovarian cancer, breast cancer and non-small cell lung cancer [4,5]. Ursodeoxycholic acid exerts a good pharmacological activity in primary biliary cirrhosis. Andrographolide (AP), known as a natural antibiotic, is commonly applied in treating dysentery, bacterial and viral upper respiratory tract infections without drug resistance [6]. Artemisinin (ART) has saved millions of lives worldwide due to its excellent anti-malarial activity. Youyou Tu, a Chinese medicinal botanist, was awarded the 2015 Nobel Prize in Physiology or Medicine for her significant effort in extracting ART from *Artemisia annua* Linn.

Although plenty of T-APIs in the early stage of development have demonstrated good pharmacological activities, they suffer from poor physicochemical properties, such as low aqueous solubility, poor stability, high hygroscopicity and erratic bioavailability (Table 1). As a result, the overwhelming majority of T-APIs thwart their success as new chemical entities for numerous clinical indications [7]. Only less than 1% of T-APIs can be eventually commercialized [8] and documented in the latest *Chinese Pharmacopoeia* (CP) 2020 edition. This extremely low percentage undoubtedly causes huge economic and resource losses to drug research centers and enterprises. In addition, the therapeutic performances of existed T-APIs were not fully maximized by their poor physicochemical properties.

The physicochemical properties of an API have a direct impact on its processing, delivery and therapeutic performance [10]. Currently, various formulation strategies have been utilized to improve the drug absorption and thus bioavailability, including milling [11]; self-emulsification [12]; solid dispersion [13]; hot melt extrusion [14]; inclusion complex [15]; nanoparticles [16]; liposomal formulations [17]; chemical modifications; and formation of other crystalline solids such as salts, hydrates and polymorphs; however, there are some limitations for each strategy. For example, salt is not practical for those very weakly ionized or nonionized compounds. Some APIs such as terpenoids are also not suitable for salt formation due to pH-dependent degradation [18]. In addition, many salts exhibit strong hygroscopicity, which is prone to deliquescence or mildew upon moisture sorption, thus affecting the drug quality (e.g., vinblastine sulfate). Polymorphs and hydrates are virtually governed by the intrinsic properties of drugs, and thus their formation is not guaranteed, and it is hard to be predicted beforehand. The limitation of chemical modification is mainly from a regulatory perspective as the modified drug is considered as a new drug entity that requires extensive evaluation on safety and efficacy.

**Figure 1:**
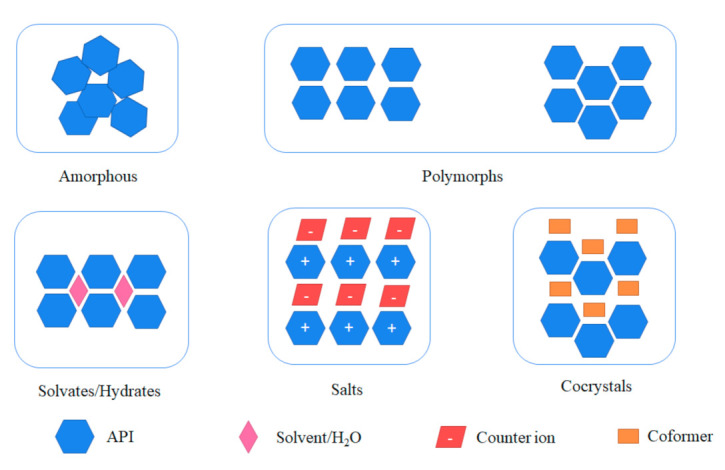
Different solid-state forms of an API.

Pharmaceutical cocrystals provide a superior alternative to amorphization and formation of other crystalline solids including salts, hydrates and polymorphs (Figure 1). Unlike salts, cocrystals are applicable to non-ionized compounds [19] with more flexibility in choosing suitable coformers for cocrystallization. In contrast to amorphous solids, cocrystals can render a higher thermodynamic stability but possible to remain solubility advantage. In general, compared to solvates and polymorphs, cocrystals normally present a superior physical stability as cocrystal coformers are seldom likely to volatilize or sublimate, while it is common for solvates and polymorphs to undergo undesirable phase transformations such as desolvation or polymorphic transition. However, it is worth mentioning that some cocrystals may also exist polymorphs or solvates. In addition to the solid states mentioned above, eutectic mixtures [20] could also enhance solubility, dissolution rate and oral bioavailability of poorly water-soluble drugs [21]. From the viewpoint of thermodynamics, it is much easier to form eutectics than cocrystals, but their relatively low thermal stability and unpredictable stoichiometry may cause problems during drug development. Temperature-composition phase diagram and powder X-ray diffraction (PXRD) are useful to distinguish cocrystals from eutectics. Furthermore, cocrystals offer unique merits over other techniques to modify physicochemical properties of drugs by non-covalent interactions without altering the pharmacological properties [22]. Cocrystals are currently considered as drug product intermediates rather than new APIs or pro-drugs under the regulatory framework of the U.S. Food and Drug Administration (FDA) [23].

Cocrystallization can be defined as combining two or more discrete molecules together in a fixed stoichiometric ratio in a crystalline lattice via non-covalent interactions, such as hydrogen bonds, π–π interactions and halogen bonds [24]. Cocrystal engineering involves designing extensive supramolecular synthons in crystal lattice and ultimately gives rise to formation of cocrystals [22]. The primary hurdle of cocrystal screening is identification of appropriate coformers, and the trial-and-error approach is still the main strategy as knowledge of synthons cannot guarantee the formation of cocrystals [25]. In order for the screening efficiency to be improved, various strategies have emerged for cocrystal screening, such as p*K*_a_ rule, Hansen solubility parameters calculation [26] and virtual screening techniques [27] (conductor-like screening model for real solvents (COSMO-RS) [28] and quantitative structure-activity/property relationships (QSAR/QSPR) [29]). In the COSMO-RS approach, the measurement of cocrystallization tendency depends on the excess enthalpy between the API-coformer mixture relative to the pure components. For example, COSMO-RS was used for the cocrystal screening of dicarboxylic acids with phenylpiperazine derivatives [30], and QSAR/QSPR descriptors were utilized for phenolic acid cocrystal screening with predicted physicochemical properties and biological activities [29]. These techniques are beneficial to the development of pharmaceutical industry for rapid cocrystal screening and thus drug development. Cocrystallization is a promising approach with great applicability and flexibility, and flexible dosing regimens are possible for T-APIs as their dosing control are less strict than drugs, allowing more flexibility in T-API cocrystallization. In particular, without altering its chemical structure and function, cocrystallization can manipulate physicochemical and biopharmaceutical properties of APIs for desired therapeutic outcomes, including solubility, dissolution rate, hygroscopicity, taste, chemical stability, tabletability and bioavailability [31]. Cocrystals hold great promise to improve the therapeutic efficacy of drugs and have a profound impact on pre-clinical research and dosage form design of drugs.

Some pharmaceutical cocrystal formulations, i.e., valproate semisodium (Depakote^®^), chloral betaine (Beta-Chlor^®^), escitalopram oxalate (Lexapro^®^) and caffeine citrate (Cafcit^®^) have been commercialized in different countries. In 2015, a drug–drug cocrystal Entresto^®^ containing a hemi-pentahydrate form of sacubitril’s sodium salt and the disodium salt of valsartan was approved by the U.S. FDA for the treatment of heart failure. It is worth noting that the cocrystal can cut down the dosing frequency of valsartan due to a rise in the bioavailability upon cocrystallization [32].

According to the Cambridge Structural Database (CSD) 2020, the number of cocrystals involving TCM ingredients has been significantly increasing in the last decade [33]. While the modifications of pharmaceutical properties through cocrystal engineering have been extensively reported frequently, the designs and applications of T-API cocrystals are still in an early research stage without any approved TCM cocrystal in current drug market.

In this review, the pharmaceutical applications and underlying mechanisms of cocrystal engineering in manipulating physicochemical and biopharmaceutical properties of T-APIs are systematically discussed. This review also provides a collective summary and an insight of the potentials and challenges of applying cocrystal engineering into new drug development of problematic T-APIs.

## 2. The Physicochemical Properties of Flavonoids, Terpenoids, Alkaloids, and Phenolic Acids in T-APIs

T-APIs generally originate from Chinese traditional oral decoctions. Compared to other delivery routes, oral administration is the most common drug delivery route due to its greater convenience, lower pain, higher patient compliance and reduced risk of cross-infection, etc. [34]. Unfortunately, most of the T-APIs have poor physicochemical properties, such as low solubility, poor stability and high hygroscopicity. The physicochemical problems of the main structural types of T-APIs (flavonoids, terpenoids, alkaloids and phenolic acids) are shown in Table 1 and Table 2. Some T-APIs with poor bioavailability have to be formulated for intravenous injection, leading to some drawbacks such as high cost, inconvenience in terms of use, and some adverse reactions [35].

### 2.1. Flavonoids

**Figure 2:**
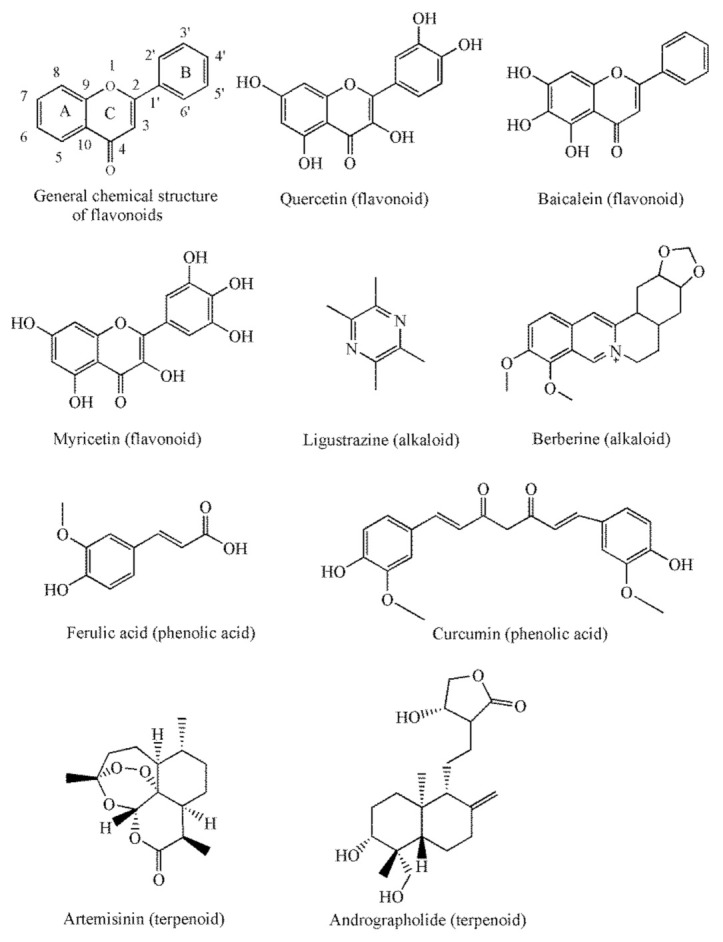
The representative compounds of flavonoids, alkaloids, phenolic acids and terpenoids in this review.

Flavonoids represent a group of natural phenolic compounds that are characterized by a phenyl benzo (γ) pyrone-derived structure consisting of two benzene rings (A and B) linked via pyrane rings (C) (Figure 2) [3]. The molecular structures of bioflavonoids contain phenolic hydroxyl groups.

The existence of carbonyl and phenolic hydroxyl groups in the flavonoid leads to the formation of intramolecular and intermolecular hydrogen bonds, which makes the molecules tightly linked [37]. In addition, π–π interaction of aromatic rings contributes to the binding of molecules, and thus flavonoids generally present a planar structure. For instance, the double bond between positions 2 and 3 of flavones and flavonols are amenable to form the planar structures, leading to tight molecular arrangement, and are consequently hard to be penetrated by solvent molecules [38,39]. This causes their low aqueous solubility and poor bioavailability. For example, baicalein (BAI), a flavonoid compound, is practically insoluble in water [40]. Myricetin, a typical flavonol with planar structure, showed only 9.62% oral bioavailability in rats, likely owing to its low aqueous solubility of 16.60 μg/mL [41]. The multiple phenolic hydroxyl groups contained in quercetin whose aqueous solubility is only 7 mg·L^−1^, are easy to form hydrogen bonds between molecules, exhibiting high lattice energy [42]. Therefore, solubility is an important consideration for the development of flavonoids as drugs [3].

Aside from solubility problems, flavonoids possess other disadvantages as well. For example, baicalein molecules are combined into dimers by hydrogen bonds. Their dimers are arranged in parallel and stacked together through π–π interactions, and as a result represent a very dense structure. Baicalein (BAI) is not prone to plastic deformation due to the π–π interactions, resulting in deficient tabletability.

Flavonoid hydrates and glycosides suffer from different problems than flavonoids. Some flavonoids can readily form hydrates via formation of intermolecular hydrogen bonds between their polar groups and water molecules. Hydrates adopt a more planar conformation than anhydrous forms, which allows them to pack more closely by strong π–π stacking interaction, thus resulting in a greater chemical stability in the crystal lattice [43]. Therefore, aqueous solubilities of hydrates, such as quercetin dihydrate, are lower than their anhydrous forms [43]. Although the aqueous solubilities of flavonoid glycosides are higher than that of aglycones, it is difficult for them to be absorbed passively through the small intestine. The existence of multiple hydroxyl groups in the molecular structure (such as hydrogen bonding) is the reason for the poor passive diffusion absorption [44].

### 2.2. Alkaloids

Alkaloids are a class of naturally occurring organic nitrogen-containing bases, which widely exist in medicinal plants. According to the structures of parent nucleus, alkaloids are mainly divided into five types, namely, isoquinoline, piperidine, scopoletin, indole and organic amine alkaloids.

Most alkaloids exhibit physicochemical problems, such as poor aqueous solubility, low bioavailability and a distinct bitter taste [45]. For instance, berberine (BB), a quaternary protoberberine isoquinoline alkaloid, is slightly soluble in water due to its strong hydrophobic functional groups, including isoquinoline, phenyl ring nucleus and oxymethyl side chain. Moreover, in vivo pharmacokinetic studies showed that the apparent permeability co-efficient cross the intestinal tissue was in the order of only 10^−7^ cm/s [46]. Berberine chloride (BCl), a salt form of berberine, showed temperature-dependent solubilities of 5.27 ± 0.29 and 8.50 ± 0.40 mM at 25 ℃ and 37 ℃, respectively [47]. Ligustrazine (TMP) is insoluble in cold water, and its pyrazine ring makes its structure planar [48].

In addition, some alkaloids have shown poor thermal stability or strong hygroscopicity. For example, TMP is prone to sublimate at 22 °C. Four oxygen atoms in the structure of BCl could easily interact with external water molecules as hydrogen bond acceptors and form monohydrate, dihydrate and tetrahydrate under different humidities.

### 2.3. Phenolic Acids

Phenolic acids have multiple phenolic hydroxyl substitutions on the benzene ring, and therefore are photosensitive and unstable. For example, some phenolic acids are prone to degradation, such as salvianolic acid B and curcumin [49]. Some phenolic acids possess low aqueous solubilities, such as olanolic acid, rhubaric acid, ferulic acid and curcumin.

Ferulic acid is one of the effective components of *Ferula sinkiangensis* K. M., *Angelica sinensis*, *Ligusticum chuanxiong* hort and other TCMs. Ferulic acid is soluble in hot water but slightly soluble in cold water. Two ferulic acid molecules can form carboxylic acid dimers through hydrogen bonds, and these dimer synthons are connected to each other through O–H (phenolic) ⋯O (carboxyl) hydrogen bonds to form a flat aqueous solubility sheet-like arrangement, which are further filled with π–π interactions as well as various C–H⋯O interactions between the benzene ring and the C–C region [50]. The salt form of ferulic acid, namely, sodium ferulate, shows better aqueous solubility than that of ferulic acid. However, after oral administration of sodium ferulate, it showed shorter peak time and faster elimination in humans (t_max_ is about 25 min, t_1/2_ is about 50 min), and thus multiple administration is needed to maintain the effective blood drug concentration for a period of time, which significantly hinders its clinical use.

Curcumin is a natural bioactive compound extracted from turmeric’s rhizome of the family Zingiberaceae. Curcumin, with both intramolecular and intermolecular hydrogen bonds, has a poor solubility in water (8.7 mg/L) [51]. There was a report claiming that the blood concentration of curcumin after 1 h was only 4.18 ng/mL after orally taking two soft capsules containing 70 mg curcumin in total, indicating its poor bioavailability [52].

### 2.4. Terpenoids

Terpenoids are the largest group, with most diverse structures derived from natural sources and widely distributed in many herbal plants. Many terpenoids have been proven to be the main active compounds in TCMs, such as artemisinin (ART), AP, ginkgolide, paeoniflorin, paclitaxel, camphor, linalool, menthol and ginsenoside.

According to isoprene numbers, terpenoids can be divided into hemi-, mono-, sesqui-, di-, tri-, tetra- and polyterpenes. In addition to the terpene forms, it also exists in the form of various oxygen-containing derivatives, such as terpene alcohols, terpene aldehydes, terpene ketones, terpenic acids, terpene esters and terpene glycosides.

Terpenoids are often structurally characterized by abundant carbon skeletons. Most terpenoids possess poor aqueous solubilities, which leads to poor drug absorption and thus low bioavailability when orally administered. On the other hand, terpenoids could easily penetrate across cell membrane due to their lipophilic properties. In general, most terpenoids are classified as biopharmaceutics classification system (BCS) class II drugs characterized by low solubility and high permeability that is rate-limiting to oral absorption [53]. It is very important to improve the solubility and oral bioavailability of poor water-soluble terpenoids and consequently increase their pharmacological efficacy. In addition, some terpenoids are sensitive to strong acid or basic conditions.

Artemisinin (ART) has some derivatives such as dihydroartemisinin, artemether and artesunate. Although famous for their excellent anti-malarial activity, their aqueous solubilities are extremely low. Aqueous solubilities of ART and 11-azartemisinin (11-AZA) were 0.0076 and 0.025 g/100 mL at 25 °C, respectively, showing their extremely low bioavailability when taken orally [54]. In addition, frequent drug administration is needed due to their rapid metabolism and short half-life [55]. ART, a sesquiterpene lactone, is an orthorhombic crystal under common conditions. Four independent ART molecules are arranged in a unit cell, and multiple unit cells are packed into regular layered network closely by non-covalent forces, resulting in its poor aqueous solubility [56]. Moreover, few polar groups and complex carbocyclic ring in ART are likely to help to decrease its aqueous solubility.

Andrographolide (AP) is a diterpene lactone compound extracted from Andrographis paniculata, showing a white square prism or flaky crystal. AP is known as a widely used natural antibiotic, while it is practically insoluble in water (3.16 ± 1.2 mg/L). Two AP molecules form an asymmetric unit by C–H⋯O hydrogen bonds [57]. Commercial AP tablets and capsules have the disadvantages of slow dissolution and onset. The structural modification compounds should solve the slow dissolution and onset problem while increasing toxic and side effects [45].

## 3. Design of T-API Cocrystals

Table 3 shows the supramolecular interactions formed in the reported T-API cocrystals. From the perspective of chemical structures, alkaloids, flavonoids, phenolic acids and phenolic acids have their own characteristic carbon skeleton or landmark functional groups, such as amino, amide, hydroxy and carboxyl groups. Coformers can be selected to prepare cocrystals by forming supramolecular interactions with APIs through intermolecular recognition based on knowledge of geometries and preferred orientations of existing intermolecular interactions [58]. Synthons are the common non-covalent intermolecular interactions of specified geometries identified in the literature that make up the structural units within a supramolecular structure [58]. When an API has been selected for pharmaceutical cocrystallization, a pharmaceutically acceptable and non-toxic conformer should be chosen. This limits the selection scope of coformers within those that have been approved for consumption by humans, for example 216 pharmaceutical excipients and compounds classified as generally recognized as safe (GRAS) for use as food additives by the U.S. Department of Health and Human Services [59]. Cocrystallization of two or three APIs has also been designed as a combination product to offer potentially synergistic effects.

### 3.1. Flavonoids

**Figure 3:**
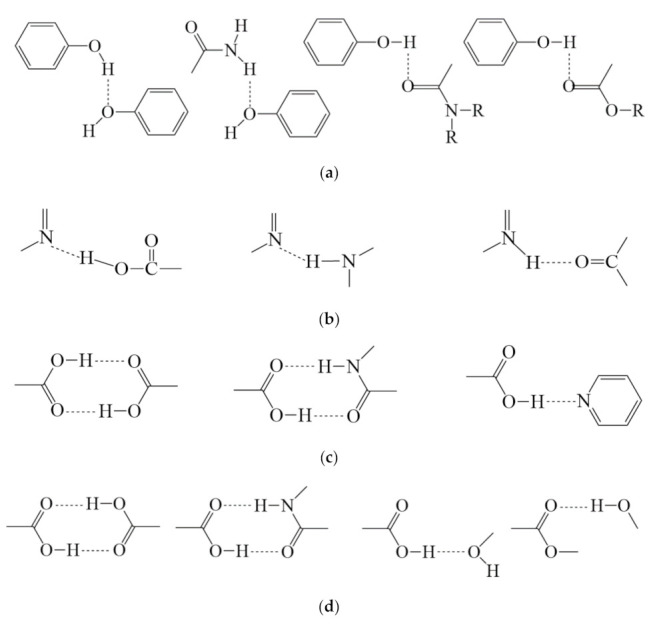
Typical hydrogen bounded elements in pharmaceutical cocrystals observed in (**a**) flavonoids, (**b**) alkaloids, (**c**) phenolic acids and (**d**) terpenoids.

Active hydrogens in multiple hydroxyl groups of flavonoids usually act as a hydrogen bond donor, forming hydrogen bonds with ligands of oxygen and nitrogen atoms that have lone pair electrons or conformation. The ligands of flavonoid cocrystals are mostly compounds containing amino or amide groups (e.g., nicotinamide, isonicotinamide), or hydroxyl or carboxyl groups (i.e., picolinic acid, L-proline). Typical examples of synthons are shown in Figure 3a.

**Figure 4:**
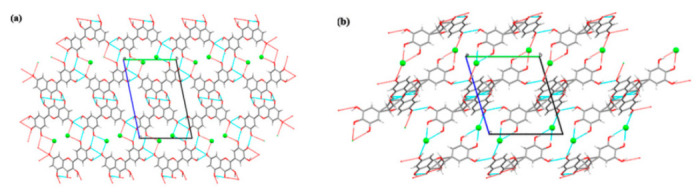
(**a**) Two-dimensional network formed between chloride anion and myricetin moieties via O–H⋯O and O–H⋯Cl^–^ interactions viewed along the a-axis (capped stick model). (**b**) Two-dimensional network formed between chloride anion and dihydromyricetin moieties via O–H⋯O and O–H⋯Cl^–^ interactions viewed along the a-axis (capped stick model) [64] (with permission from [64]; Copyright 2019 American Chemical Society).

Furthermore, the active hydrogen atom in the phenolic hydroxyl group of flavonoids is weakly acidic and capable of salt cocrystals formation with certain hydrochlorides or chlorides, such as berberine chloride–myricetin (BER-MYR) salt cocrystal and berberine chloride–dihydromyricetin (BER-DMY) salt cocrystal. The formation of these two cocrystals was mainly contributed by the intermolecular interaction of O–H⋯Cl^–^ formed between myricetin and dihydromyricetin with chloride ions (Figure 4), indicating that the phenolic hydroxyl groups of flavonoids played an important role in the formation of salt cocrystals [64].

As most flavonoid glycoside compounds are highly water-soluble, their cocrystallization applications are less explored in the literature. There was only one report that claimed the successful formation of rutin–carbamide and rutin–polyethylene glycol cocrystals currently [112]. 

### 3.2. Alkaloids

The lone electron pair on the nitrogen atom in many alkaloids is able to accept protons. If some coformers containing hydroxyl and carbonyl groups have small molecular weight and space volume (especially small molecular organic acids), they can easily form N–H⋯O or other similar hydrogen bonds to obtain cocrystals. Therefore, alkaloids could serve as coformers to each other. The typical hydrogen-bonding synthon structure in alkaloid cocrystals is shown in Figure 3b. For example, in the theophylline-nicotinamide cocrystal prepared by Lu et al., the N of theophylline and the N–H of the amide bond of nicotinamide formed a strong hydrogen bond, while the C=O of nicotinamide formed a slightly weaker hydrogen bond with the H of theophylline [113]. In addition, some alkaloids contain nitrogen positive ions, which can be combined with some anions, such as chlorine ions of hydrochloric acid, to produce a charge-assisted effect to form a cocrystal salt by hydrogen bonding.

**Figure 5:**
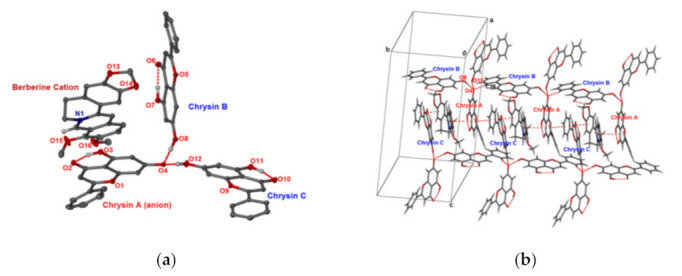
(**a**) ORTEP plot of berberine–chrysin cocrystal with 35% probability level. H atoms on C were omitted for clarity. Dashed lines represent hydrogen-bonding interactions. (**b**) Supramolecular structure resulting from C–H⋯O and π–π interactions [66] (adapted with permission from [66]; Copyright 2018 American Chemical Society).

Berberine chloride (BCl) is used to form cocrystals or cocrystal salts with coformers of polybasic organic acids such as fumaric acid, citric acid, pyromellitic acid and succinic acid. In the structure of BCl-fumaric acid cocrystal, one FA molecule connects with two BCl molecules through O–H⋯Cl hydrogen bonding and π–π stacking interaction. Berberine saccharine (BbSac) and berberine acesulfame (BbAs) salt cocrystals were obtained via the anion exchange reaction [114]. Only weak C–H⋯O hydrogen bonds without classic hydrogen bonds can be found to stabilize the crystal structures of both salts. A salt cocrystal between berberine and chrysin was also reported [66]. Crystal structure analysis demonstrateed that charge-assisted strong hydrogen bonding interactions between phenolic anions and hydroxyl groups of chrysin dominated the structure. Moreover, the ring structure of berberine participated in the π–π conjugation with the chrysin C ring structure (Figure 5).

**Figure 6:**
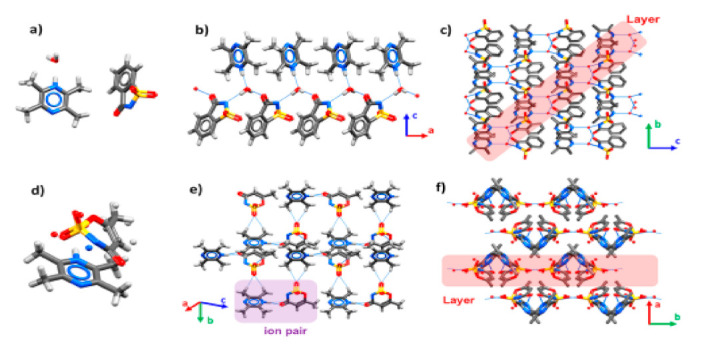
Crystal structures of TMP-SAC (**a**–**c**) and TMP-ACS (**d**–**f**). The blue lines represent hydrogen bonds [31] (with permission from [31]; Copyright 2020 American Chemical Society).

Ligustrazine–saccharine (TMP-SAC) and TMP–acesulfame (TMP-ACS) salt cocrystals were prepared by Hu et al. [31]. The active hydrogen ion (H^+^) on SAC was transferred to TMP in the TMP-SAC cocrystal. TMP+, SAC– and water molecule formed a long molecular chain through hydrogen bonding interactions of O–H⋯O=C, O–H⋯N^–^ and N^+^–H⋯O. Adjacent chains were assembled into 2D layers along the (0–11) plane by S=O⋯H–C, and each layer was stacked to form a stable 3D structure via S=O⋯H–C. In the TMP-ACS cocrystal, the active hydrogen ion (H^+^) on ACS was transferred to TMP. The TMP+ and ACS– ion pairs mainly acted as charge-assisted hydrogen bonding through N–H⋯O=C. Neighboring ion pairs further interacted through S=O⋯H–C to form a planar 2D layer. These layers were stacked to form a 3D stacking structure, which was stabilized by the weak hydrogen bonds between adjacent layers. The sublimation trend of TMP-ACS was much lower than the commercial salts such as TMP phosphate monohydrate (TMP-Pho) and TMP hydrochloride dihydrate (TMP-HCl) (Figure 6).

Wang et al. obtained organic supramolecular cocrystals through combining TMP with different acid coformers, such as 1,4-cyclohexanedicarboxylic acid, 6-hydroxy-2-naphthoic acid, 2,6-pyridinedicarboxylic acid, 2,6-dihydroxybenzoic acid, 3-nitrophthalic acid, o-phthalic acid and 3-hydroxybenzoic acid [115]. These cocrystal structures were assembled without charge transfer, and composed of classic O–H⋯O, O–H⋯N, C–H⋯O and π–π stacked non-covalent interaction to form 2D or 3D supramolecular structures. Meanwhile, the classical O–H⋯N and weak C–H⋯O interacting carboxyl/pyrazine supramolecular heterozygotes R^2^2 (6) and R^2^2 (8), etc., participated in the construction of hydrogen-bonded supramolecular networks [115].

### 3.3. Formation of Cocrystals of Phenolic Acids

Phenolic acids are widely distributed in nature, especially in many common Chinese medicines, such as *Lonicera japonica*, *Angelica sinensis*, *Ligusticum chuanxiong* Hort and *Salvia miltiorrhiza* Bge. Representative compounds, such as ferulic acid, curcumin, oleanolic acid and salvianolic acid B, usually have a wide range of physiological activities, including anti-oxidant, anti-inflammatory, cardiovascular protection and anti-bacterial properties. Phenolic acids have phenolic hydroxyl groups and carboxyl groups in their structures, which are capable of forming cocrystals or salt cocrystals through hydrogen bonding, conjugation, or charge-assisted conjugation with other compounds containing carbonyl groups, hydroxyl groups and nitrogen compounds, especially some basic molecules. Figure 3c shows several examples of typical synthesizers.

Xu et al. combined curcumin and lysine in a 1:1 stoichiometric ratio to form a cocrystal [116]. The oxygen atom in the phenolic hydroxyl group of curcumin is highly electronegative, and the surrounding steric hindrance is small. Thus, it can act as a hydrogen bond donor to attach to the guanidine group and amino group in lysine, which acts as the hydrogen bond acceptor, forming a cocrystal by O–H⋯N hydrogen bonds. Interestingly, there were two crystal forms of curcumin–lysine cocrystal. The unit cell of a-type cocrystal containing two molecules of curcumin and lysine was not linked with adjacent unit cells, and thus stacked to form spherulites. The unit cell of b-type cocrystal contained three molecules of curcumin and lysine, which was connected with adjacent unit cells through partial hydrogen bonds, thus forming ribbon-like crystals.

### 3.4. Terpenoids

Small molecule compounds containing carboxyl groups and hydroxyl groups are suitable coformers for the preparation of terpenoid cocrystals. These small molecules could easily access some terpenoids with large space and complex carbon skeleton and form strong O–H⋯O hydrogen bonds with the oxygen-containing functional groups of terpenoid compounds. For example, the hydroxyl groups in AP can be complementary to coformer molecules containing –C=O, –COOH and –OH functional groups, resulting in strong O–H⋯O hydrogen bonds to form cocrystals. In addition, amides or amines can also be used as coformers to form cocrystals through N–H⋯O hydrogen bonds. Some cocrystals of sesquiterpenes (i.e., artemisinin) [18], diterpenes (i.e., rographolide) and triterpenes (i.e., oleanolic acid) were reported. Figure 3d shows several examples of typical synthesizers.

ART is not appropriate for salt formation due to its lack of ionizable sites and sensitivity of the endoperoxide moiety to acidic and basic conditions [18]. Moreover, solvate is not a suitable form for ART, owning to its low stability, whereas the cocrystal form could tackle the above problems. Karki et al. prepared a 2:1 artemisinin–resorcinol (ART-RES) cocrystal and 1:1 artemisinin–orcinol (ART-ORC) cocrystals. ART-RES cocrystal formed an O–H⋯O hydrogen bond from the carbonyl group of ART and hydroxyl group of RES, constituting a trimer unit of one molecule of ART and two molecules of RES. Then, the trimer unit formed a layered structure with a certain twisted surface through extensive C–H⋯O contact network interaction. A basic unit of R^2^2 (8) in ART-ORC cocrystal was firstly formed with a certain distortion from O–H⋯O hydrogen bond between hydroxyl of ORC and carbonyl group of ART, and then connected into molecular chains. Furthermore, C–H⋯O interactions existed between carbon atoms of ORC and bridged oxygen atoms, and the resulting tape was further assembled through interactions of C–H⋯π and C–H⋯O [18].

11-Aza-artemisinin (11-AZA) is a derivative of ART. A variety of 11-AZA cocrystals were prepared with different coformers, such as salicylic acid, benzoic acid, succinic acid and pimelic acid, exhibiting high solubility. Strong C=O⋯H–O bonds supplemented by weaker NH⋯O=C bonds form the basic unit of R^2^2 (8), including lactams and acid hetero-molecule, in most of the 11-AZA cocrystals [54,117].

## 4. Preparation of Cocrystals

Compared to conventional chemical synthesis, cocrystallization could be performed under relatively mild reaction conditions. The preparation methods of cocrystals can be broadly categorized as solid-state and solution-based. Table 4 summarizes the methods reported for cocrystallization of T-APIs.

Solution cocrystallization means dissolving APIs and coformers in a certain stoichiometric ratio in a suitable solvent and subsequently removing the solvent to induce supersaturation of solutes for cocrystal formation. Solution cocrystallization consists of solvent evaporation (slow evaporation and rapid solvent removal), reaction cocrystallization, slurry and antisolvent diffusion. It relies on stronger intermolecular interactions between APIs and coformers rather than intramolecular interactions to enable cocrystal formation. For solution cocrystallization, prevention of the least soluble component from sole precipitation is needed, and choosing components with congruent solubilities could improve the phase purity of cocrystals [92]. Thus, in addition to selection of APIs and conformers, judicious selections of a solvent system, a stoichiometric ratio and crystallization temperature are also important [118]. The analysis of cocrystal structures from the CSD suggests that components with similar shapes and polarities are favorable to cocrystallize with each other [119].

Solid state grinding is a method that mixing APIs with coformers in mortar or ball mill to prepare cocrystals under mechanical power, and its mechanism depends on the molecular mobility and complementarity of APIs and coformers.

Liquid-assisted grinding refers to the addition of solvents in very small amount to a dry solid prior to the initiation of milling. The solvent has a catalytic role in assisting cocrystal formation that should persist during the grinding process. Compared with the neat method, the liquid-assisted method is more efficient in cocrystal formation. With the increase of the solvent added to the grinding medium, the kinetics of cocrystal formation tends to increase [120], but this has not been confirmed yet. The liquid component is thought to accelerate reaction kinetics by wetting the solid surface. Liquid-assisted grinding has been reported in many different forms.

Except from the supercritical fluids, aforementioned methods, high throughput crystallization and spray drying are also applied for pharmaceutical cocrystallization [121,122].

**Table 4:** The preparation methods of T-API cocrystals.

Preparation Methods	Drug	Co-Former
Slow evaporation	Baicalein	Caffeine [123]
	Berberine	Phthalic acid * [65]
	Curcumin	2-Aminopyridine * [124], 2,5-Dihydroxybenzoic acid * [125]
	Rutin	Carbamide [112], polyethylene glycol * [112]
	Puerarin	Lornoxicam [86]
	Ferulic acid	Nicotinamide [100]
	Ursolic acid	Ethylenediamine * [107], piperazine [108]
	Oleanolic acid	Ethylenediamine * [126], piperazine [127]
	Ligustrazine	Saccharine [128], febuxostat [129]
Rapid solvent removal	Curcumin	Isoniazid [90], hydroquinone * [91], phloroglucinol [92]
Slurry	Berberine chloride	Fumaric acid [62], myricetin [64], dihydromyricetin [64]
Recrystallization	Matrine	Salvianolic acid B [130]
Supercritical fluids	Resveratrol	Curcumin [104]
Antisolvent precipitation	Ursolic acid	Metformin [109], arginine [109], lysine [109], N-methylglucamine [109]
Neat grinding	Curcumin	Trimesic acid [95]
Solvent assisted grinding	Berberine chloride	Citric acid [60], ibuprofen [61], fumaric acid [62]
	Ligustrazine	Ethinylestradiol [131]
	Baicalein	Nicotinamide [69]
	Celastrol	Threonine [132], phenylalanine [132], L-tyrosine [132]

Note: * indicates belonging to non-GARS or non-drug compounds.

## 5. Modifications of Physicochemical Properties of API of TCMs through Cocrystal Engineering

Physicochemical properties are crucial properties for consideration during the development of new dosage forms [22]. Cocrystals offer advantages over other techniques to modulate different physicochemical properties of drugs without altering the pharmacological properties by non-covalent interactions [22]. Herein, we summarize the applications of cocrystal engineering of T-APIs on the basis of some key physicochemical properties, such as solubility, stability, dissolution performance and bioavailability (Table 5).

### 5.1. Stability

Stability is the most important parameter to be studied during drug development. The drug self-life mainly depends on the physicochemical stabilities [22]. The following properties are the detailed aspects to be considered for stability testing.

#### 5.1.1. Thermal Stability

Sublimation is a critical physical phenomenon underlying a number of important industrial processes, such as freeze drying, purification, and dye sublimation printing [31]. If APIs shows fast sublimation at ambient environments, uncontrolled loss of drug during manufacturing and storage would occur. As a result, it will lead to inaccurate dose and suboptimal therapeutic outcomes. This problem will be aggravated if there is any unit operation involving heating (e.g., drying and milling) during manufacturing. Special packaging or formulation may be required to address volatility of such drugs to maintain stability on storage. In the examination of the thermal stability, differential scanning calorimetry (DSC) and thermal gravimetric analysis (TGA) are two important techniques for capturing the change of melting points and weight loss, respectively [133]. For example, TMP will sublimate at 22 °C, and commercial TMP hydrochloride and TMP phosphate also tend to sublime readily [31]. It brings challenges for the storage of these compounds and dosage form production. TMP–acetylsulfonamide (TMP-ACS) salt cocrystal was prepared by Hu et al. to improve the thermal stability of TMP, and it exhibits a significantly reduced sublimation tendency and hygroscopicity compared to the TMP hydrochloride and phosphate salts [31].

Wang et al. reported the formation of several TMP cocrystals with improved stabilities when cocrystallizing with various coformers, including 1,4-cyclohexanedicarboxylic acid, 6-hydroxy-2-naphthoic acid, 2,6-dihydroxybenzoic acid, o-phthalic acid, 2,6-pyridinedicarboxylic acid, 3-hydroxybenzoic acid, and 3-nitrophthalic acid [115]. The sublimation temperature of these cocrystals was found to be above 100 °C, which is suitable for the actual storage environment and higher than that of TMP hydrochloride (75 °C), indicating its better thermal stability than TMP hydrochloride. For example, there was a sharp weight loss ending at 270 °C for TMP-2,6-dihydroxybenzoic acid cocrystal, 250 °C for TMP-3-hydroxybenzoic acid cocrystal and 320 °C for TMP-1,4-cyclohexanedicarboxylic acid cocrystal, respectively. All cocrystals were air stable and could retain their structural integrity at ambient conditions for a considerable length of time [115]. In addition, a literature showed that berberine–fumaric acid (BBC-FA) cocrystals highly improved the stability of BBC at high humidity and temperature. The decomposition temperature of BBC-FA cocrystal was 224.07 °C, which was higher than that of BCL dihydrate (BCD) and BCl tetrahydrate (BCT) (181.40 and 176.33 °C, respectively) [62].

#### 5.1.2. Hygroscopicity

Berberine chloride (BCl) is an example with strong hygroscopicity. At relative humidity (RH) of 10%, BCl quickly absorbed water and obtained a weight equivalent to its dihydrate. The total weight gain at 95% RH was consistent with the expected water content in the tetrahydrate (16.3%). However, absorbed water in BCl–citric acid (BCl-CA) cocrystal reached less than 2% at 70% RH and 8% at 95% RH. Therefore, BCl-CA cocrystal was superior to BCl·2H_2_O in physical stability against RH changes [60]. Berberine chloride–myricetin (BER-MYR) salt cocrystal and berberine chloride–dihydromyricetin (BER-DMY) salt cocrystal showed low moisture adsorption up to 95% of relative humidity [64]. Berberine–chrysin salt cocrystal presented a low moisture adsorption, which absorbed 0.7% of water at 80% RH, and the water sorption was up to 1.6% at 95% RH. The decrease in hygroscopicity may be caused by the hydrogen bonding between the berberine molecule and its coformer, which occupied the berberine hydration site [66].

**Table 5:** Physicochemical properties modified through pharmaceutical cocrystals.

Pharmaceutical Applications	Drug	Coformer
Enhanced solubility and dissolution rate	Andrographolide	Salicylic acid [110]
	11-Aza-artemisinin	Benzoic acid [54], salicylic acid [54], succinic acid [54], heptanedioic acid [54]
	Baicalein	Isoniazid [70], caffeine [70], isonicotinamide [70], theophylline [70], betaine [71]
	Berberine chloride	Fumaric acid [62], lactic acid * [63]
	Celastrol	Threonine [132], phenylalanine [132], L-tyrosine [12]
	Curcumin	Resorcinol [93], phloroglucinol [92], Hydroquinone * [91]
	Ligustrazine	Saccharine [128]
	Myricetin	Berberine chloride [64]
	Oleanolic acid	Ethylenediamine [126], piperazine [127]
	Puerarin	Lornoxicam [86]
	Quercetin	Isonicotinamide [7], caffeine [7], theobromine dihydrate [7], betaine [71]
	Ursolic acid	Piperazine [108], ethylenediamine [107]
Hygroscopicity	Baicalein	Isoniazid [70], isonicotinamide [70], caffeine [70]
	Berberine	Chrysin [66]
	Berberine chloride	Saccharin [114], acesulfame [114]
	Curcumin	Resorcinol [93], pyrogallol [93]
	Dihydromyricetin	Berberine chloride [64]
	Ferulic acid	Isonicotinamide [100]
	Myricetin	Berberine chloride [64]
	Oleanolic acid	Ethylenediamine [126], piperazine [127]
	Quercetin	Betaine [71]
	Ursolic acid	Piperazine [108], ethylenediamine [107]
Extended release	Curcumin	Isoniazid [90]
	Piperazine ferulate	Pyrazinamide [134]
Improved tabletability	Baicalein	Nicotinamide [69], caffeine [69], isoniazid [69]
	Berberine chloride	Saccharine [114], acesulfame [114]
	Puerarin	Lornoxicam [86]
Improved thermal stability	Berberine chloride	Fumaric acid [62]
	Ligustrazine	Saccharin [31], 1,4-cyclohexanedicarboxylic acid * [115], 2,6-dihydroxybenzoic acid * [115], 2,6-pyridinedicarboxylic acid * [115], 6-hydroxy-2-naphthoic acid * [115], 3-hydroxybenzoic acid * [115]
Taste masking	Berberine chloride	Saccharin [114], acesulfame [114]
	Ligustrazine	saccharine [128]
Increased Chemical stability	Andrographolide	Salicylic acid [110]

Note: * indicates belonging to non-GARS or non-drug compounds.

#### 5.1.3. Chemical Stability

AP and AP–salicylic acid (AP-SLA) cocrystal were treated with HSO_3_^–^ solution (in the presence of HSO_4_^–^ and excess amount of Na_2_SO_3_) to simulate the biological transformation process [110]. Through analysis by LC–MS and NMR spectroscopy, AP was found to be converted to AP–SO_3_H under the action of HSO_3_^–^, while no transformation of AP–SO_3_H could be detected in the AP-SLA cocrystal. It was speculated that SLA possessed inhibitory effects on the chemical transformation of AP to AP–SO_3_H, owing to its acidic property and low pK_a_ of the –COOH group.

### 5.2. Organoleptic Properties

Bitterness is one of the greatest challenges for commercialization of berberine due to a lack of patient compliance, especially in pediatric patients. Sun et al. prepared BbAs and BbSac salt cocrystals by cocrystallizing BB with the acesulfame or sweetener saccharine to alleviate the bitter taste of BB [114].

TMP also faces the same problem of bitter taste and low oral bioavailability by the commercial phosphate salt (TMP-PHO). Sun et al. tackled these problems by forming salts with two sweeteners, acesulfame (ACS) and saccharine (SAC) [128], which effectively masked the bitter taste of TMP. Moreover, both TMP-ACS and TMP-SAC cocrystals exhibited approximately 40% higher bioavailability through reducing the absorption rate of TMP. Thus, salt cocrystal formation with both sweeteners simultaneously addressed the issues brought by the bitter taste and lower bioavailability of TMP.

### 5.3. Solubility

In contrast to amorphous forms, cocrystals can maintain thermodynamic stability in the solid state while providing solubility advantage over a drug [58].

Hong et al. manufactured myricetin cocrystals with caffeine, isonicotinamide, nicotinamide and 4-cyanopyridine [135]. The solubility of myricetin in these cocrystal forms was increased by 3–80 times in different media, and the time required to reach the maximum concentration was reduced to 10–20 min, which greatly improved the solubility of myricetin. A variety of 11-AZA cocrystals were prepared with coformers, such as benzoic acid (BEN), salicylic acid, succinic acid and pimelic acid, exhibiting significantly higher solubility than that of 11-AZA and ART. For example, the solubility of 11-AZA-BEN cocrystal was 0.11 g/100 mL, while the solubility of 11-AZA was 0.025 g/100 mL in 25 °C water [117]. The solubility of andrographolide–salicylic acid (AP-SLA) cocrystal was 12 times higher than that of AP, and intrinsic dissolution rate (IDR) was three times higher than that of AP [110]. The equilibrium solubility of the quercetin–betaine (QUE-BTN) cocrystal in phosphate buffer pH 6.8 was about four times that of quercetin dehydrate [71].

Zhu et al. prepared cocrystals of baicalein (BAI) with isoniazid, isonicotinamide, caffeine and theophylline [70]. The amide part of these coformers can easily form medium-strength hydrogen bonds with the carbonyl and hydroxyl groups of BAI to obtain cocrystals. The in vitro dissolution experiment results exhibited that all these BAI cocrystals showed a faster dissolution rate and larger apparent solubility (S_max_) than pure BAI. For example, the solubility of BAI-CAF cocrystal improved by approximately 2.5-fold and 1.5-fold in pH 2.0 and pH 4.5 buffer solutions, respectively. According to the value of AUC_0−24 h_, the relative bioavailability of BAI-CAF cocrystal improved by about 4.1-fold in comparison with that of crystalline BAI [70].

### 5.4. Dissolution Performances

After berberine (BBC) and fumaric acid (FA) were synthesized as BBC-FA cocrystals, the dissolution and stability were significantly improved [62]. The IDR of BCD and BCT were 0.3258 and 0.3214 mg·cm^−2^·min^−1^, respectively, while the IDR of BBC-FA cocrystal was 1.0397 mg·cm^−2^·min^−1^, about threefold more than that of the two hydrates.

When curcumin forms cocrystals with nicotinamide (NAM), ferulic acid, hydroquinone (HQ), p-hydroxybenzoic acid (PHBA) and L-tartaric acid (TA), the characteristic dissolution rates of various cocrystals in 40% ethanol/water solution (V:V) at 30 °C were 10.60, 6.75, 5.64, 4.43, and 2.16 times that of curcumin, respectively [136]. The amount of dissolved curcumin after 5 h (AUC _0–5 h_) was 315.1 mg·h/L for CUR, 1842.6 mg·h/L for CUR–NAM, 1256.0 mg·h/L for CUR–ferulic acid, 1059.7 mg·h/L for CUR–PHBA, 1016.5 mg·h/L for CUR–HQ and 530.7 mg·h/L for CUR–TA, respectively. High AUC value exhibited large amount of total dissolved curcumin in a given period. CUR–NAM cocrystal showed six times higher AUC and 10-fold faster IDR in comparison with curcumin.

**Figure 7:**
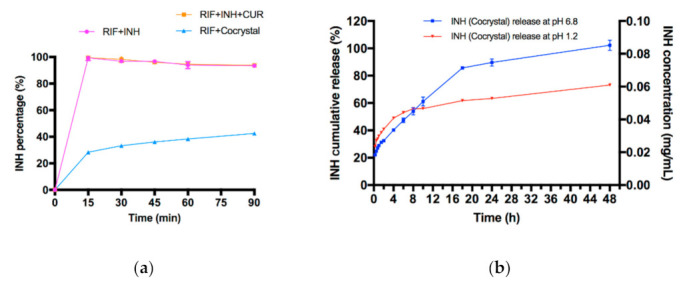
(**a**) Dissolution profiles of INH from different samples in pH 1.2 buffer solution (*n* = 3); (**b**) dissolution profile of INH from 2:1 INH-CUR cocrystal in the presence of RIF in pH 1.2 and pH 6.8 buffer solutions (*n* = 3) [90] (with permission from [90]; Copyright 2020 American Chemical Society).

Apart from improving dissolution performance, cocrystallization of T-APIs to achieve extended release profiles is a promising application as well. Xuan et al. successfully prepared an extended release form of a curcumin-isoniazid (CUR-INH) cocrystal [90]. This CUR-INH cocrystal could lower the dosing frequency of long-term usage of INH and alleviate its hepatotoxicity due to the hepatoprotective effect of CUR. In the pH 1.2 phosphate buffer, only 28% of INH was released from the CUR-INH cocrystal after 15 min, while INH was completely released from the physical mixture. Figure 7 shows that the extended release of INH was observed in both pH 1.2 and 6.8 buffers, lasting for 48 h and 24 h of INH release without using polymers, respectively. Xuan et al. explained the reduced dissolution rates of INH for two reasons: (1) CUR would block the solvation sites of INH to decrease its release rate, and (2) metastable CUR form III recrystallized from the buffers with varied sizes to cover the surface of CUR-INH cocrystals. In addition, Xuan et al. further studied the impact of cocrystal solution-state stability on cocrystal dissociation and polymorphic drug recrystallization during dissolution by three CUR cocrystals, confirming that stable CUR cocrystal required a higher supersaturation level than metastable CUR cocrystals due to the difference of intermolecular interactions [137]. Yu et al. have synthesized and characterized a novel sustained-release drug–drug ternary salt cocrystal of piperazine ferulate (PRZ-FLA) with pyrazinamide (PRA), which represents a nephropathy treatment drug and an anti-tuberculosis drug, respectively [134]. The inherent dissolution rates of ternary PRZ-FLA-PRA salt cocrystal in pH 1.2, 4.0, and 6.8 buffers were only 1/5–1/3 of PRZ-FLA, decreasing the fast elimination of FLA, which resolved the issues of short half-life of PRZ-FLA. In addition, it provides an optional cooperative treatment for the kidney injury complications and tuberculosis.

### 5.5. Bioavailability

Vasisht et al. have prepared quercetin–nicotinamide cocrystal and quercetin–picolinic acid cocrystal. The maximum solubility concentrations (S_max_) of these two cocrystals in pH 7.4 phosphate buffer were 5-fold and 10-fold that of quercetin, respectively [138]. The relative bioavailability (calculated by dividing the AUC_tot_ of the cocrystal by the AUC_tot_ of quercetin) in rats after oral administration was more than one times higher than that of quercetin. Meanwhile, these two cocrystals showed stronger DPPH free radical scavenging effects and rat erythrocyte hemolysis inhibitory effects in vitro than quercetin, which could be explained by the fact that these cocrystals have higher solubility and bioavailability and higher effective concentration accordingly, which was reflected in the enhancement of related pharmacological activities. Smith et al. synthesized four quercetin cocrystals with better bioavailability than quercetin. Among them, the quercetin–theobromine dihydrate cocrystal exhibited the highest AUC increase, which was 10 times more than that of quercetin [7].

### 5.6. Tabletability

Tabletability refers to the capacity of powders to be transformed into tablets under the compaction pressure. The tablet is an attractive dosage form since it is convenient to carry, administer and identify [139]. Other advantages include precise dosage, good storage stability, potential of controlled drug release, low production cost, and options for specialized formulations. The extremely poor compaction property of some commercial solid forms of APIs, however, is one of the main challenges for successful tablet development because the expected dose is frequently higher than 200 mg. To conquer this compaction issue, a large number of excipients are normally needed in the formulation, which will inevitably make the size of the tablets unduly large [139].

**Figure 8:**
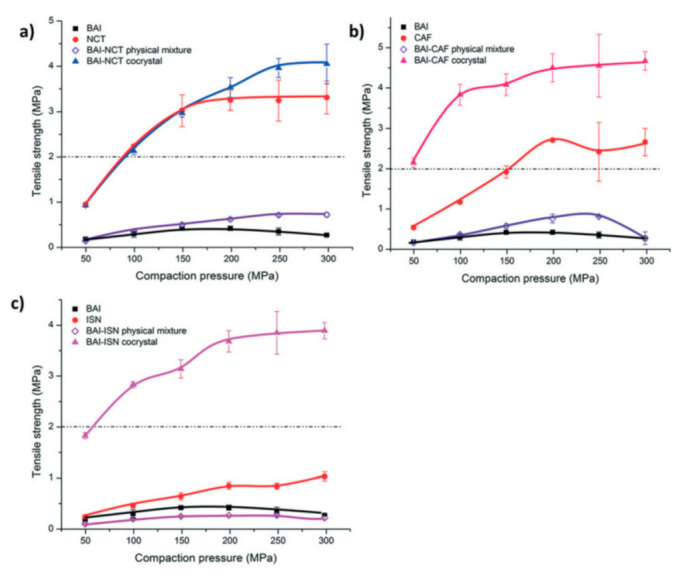
Tabletability of (**a**) BAI-NCT, (**b**) BAI-CAF, and (**c**) BAI-ISN cocrystal systems (*n* = 3). Some error bars are hidden by symbols. The dashed lines correspond to 2 MPa tensile strength [69] (Reproduced from ref. [69] with permission from the Royal Society of Chemistry).

**Figure 9:**
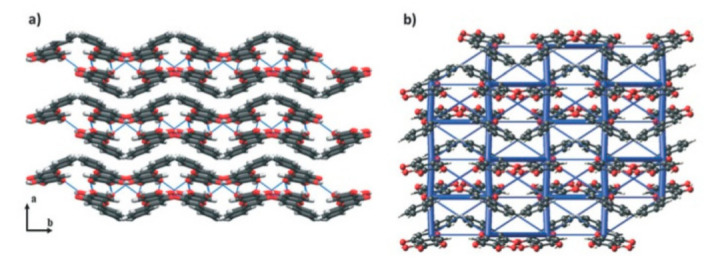
(**a**) Crystal packing diagram (hydrogen bonds are shown as blue lines) and (**b**) energy framework of BAI α form viewed into *c* axis (radius of each cylinder represents the relative strength of interaction). The threshold interaction energy is set at −5 kJ mol^−1^ [69] (Reproduced from ref. [69] with permission from the Royal Society of Chemistry).

**Figure 10:**
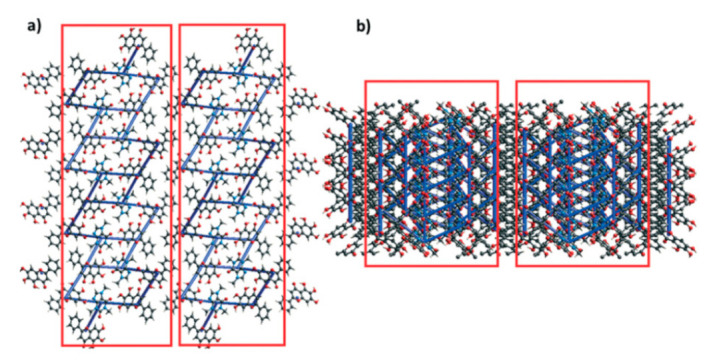
Energy framework of BAI–CAF viewed along (**a**) *b* axis and (**b**) *c* axis. The layers are boxed. The threshold interaction energy is set at −20 kJ mol^−1^ [69] (Reproduced from ref. [69] with permission from the Royal Society of Chemistry).

Liu et al. prepared BAI cocrystals with three coformers, nicotinamide (NCT), caffeine (CAF) and isoniazid (ISN) [69]. Surprisingly, all three cocrystals exhibited excellent tabletability (Figure 8), indicating that the tabletability of cocrystals was decoupled from that of coformers. The interaction energy between middle layers of BAI-CAF cocrystal was much lower than that of inner layer. Under the action of external force, BAI-CAF cocrystal showed plastic bending behavior and tensile strength more than several times higher than BAI, indicating better tableting property (Figure 9 and Figure 10) [69].

### 5.7. Others

As melting point has a correlation with aqueous solubility and vapor pressure, it is used for purity identification and characterization. It is common for a cocrystal to achieve intermediate melting point [140]. In some cases, however, a cocrystal may have a higher or lower melting point than that either of its parent components because of the significant change in crystal structure or lattice packaging. It is therefore viable to tune the melting point of a cocrystal to a required process, if appropriate cocrystal phases can be prepared for a given API.

A plenty of APIs could be served as coformers for cocrystal formation, and drug– drug cocrystal can play synergistic effects of two APIs to treat disease along with the advantage of cocrystal content more prominently. For example, berberine (BER) was made a salt cocrystal with a drug coformer MYR or DMY with similar efficacy, and both BER-MYR and BER-DMY salt cocrystals showed the synergistic anticancer effect in vitro [64]. Piperazine and TMP have circulatory improvement activities similar to ferulic acid. After administration of drug–drug cocrystal of piperazine ferulate, the mean perfusion flow of coronary artery in isolated guinea pig heart was increased by 35.83%, which was higher than that of sodium ferulate (21.7%).

## 6. Mechanisms for Modifications of Physicochemical Properties

### 6.1. Solubility

The solubility depends mainly on the solvent affinity and lattice strength. Cocrystals have the ability to improve solvent affinity and lessen lattice strength [141]. Moreover, chlorine-free acid ion can be provided by coformers to reduce common ion effect in gastric juice. Therefore, bioavailability and efficacy of a drug could be influenced by the change of its aqueous solubility.

Plenty of studies in the literature relate the solubility of cocrystals to the solubility of coformers. Coformers could affect the solvation barrier of cocrystals. This behavior is a result of a decrease in the solvation barrier for a cocrystal to an extent proportional to that of the pure API. Cocrystal solubility ratio as a function of ligand solubility for the solvent confirms that high ligand solubility equates to high cocrystal solubility [142]. Many studies have shown that some coformers with good aqueous solubility (such as nicotinamide and isonicotinamide) can significantly improve the solubility of T-APIs.

**Figure 11:**
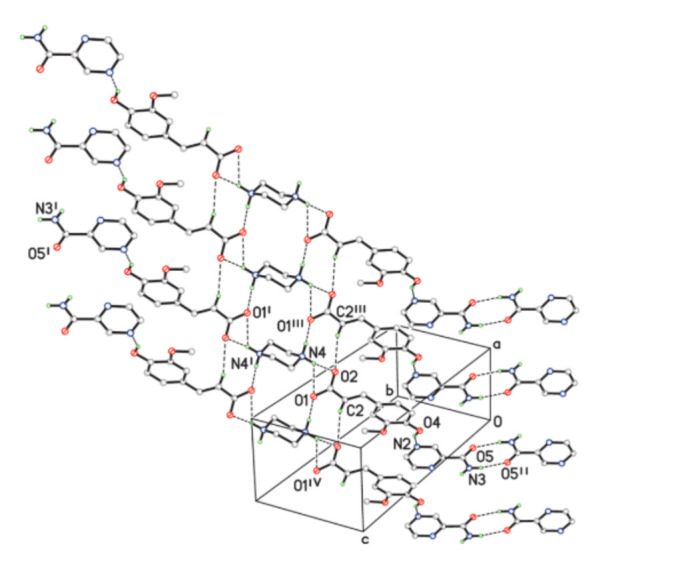
A two-dimensional classical hydrogen bonding network, in which an aromatic stacking interaction is observed between the benzene ring of FLA and the near pyrazine ring of PRA. Symmetry codes: (i) 2—x, 3—y, 1—z; (ii) –x—1, -y, -z; (iii) x + 1, y, z; (iv) x—1, y, z [134] (with permission from [134]; Copyright 2020 American Chemical Society).

Intramolecular and intermolecular hydrogen bonds, C–H⋯π interactions, and packing interactions of T-APIs constitute the hydrophobic region of the crystal structure. The introduction of appropriate coformer molecules by cocrystal technology allows T-APIs to form new structures, spatial arrangements, and hydrophilic regions, thereby improving their water solubility. For example, the maximum solubilities of quercetin–caffeine·MeOH (QUE-CAF·MeOH) cocrystal, QUE-INM cocrystal, and QUE·2H_2_O were 2.018, 1.22, and 0.267 mg/mL in ethanol/water (1:1 V/V), respectively. The solubilities of the two cocrystals were several times higher than that of QUE dehydrate [7]. In the ferulic acid–TMP cocrystal, there were one TMP molecule and two ferulic acid molecules at the crystal inversion center. The O–H⋯N hydrogen bond between the molecules and the weak C–H⋯O interaction led to the formation of supramolecular network. Compared with planar structure of ferulic acid, ferulic acid–TMP cocrystal was not coplanar with the dihedral angle of 69.45(9)° between the phenyl and pyrazine ring. Such a structure change may enable the cocrystals to provide a large space gap for water molecules, thus increasing aqueous solubility of cocrystals. The ternary salt cocrystal has a formula of (PRZ^2+^)·(FLA^–^)_2_·(PRA) _2_ revealed by single-crystal X-ray diffraction, in which the PRZ^2+^ and FLA^–^ ions form a 1D chain through the strong charge-assisted hydrogen bonds with hydrogen bond N^+^–H⋯O^–^, N–H⋯O, and C–H⋯π conjugation, and then interact with neutral PRA molecules by hydrogen bonds C–H⋯O to form a supramolecular network (Figure 11). As a result, a three-dimensional hydrogen-bonding supramolecular structure was obtained. The 3D structure of the ternary cocrystal had relatively tight intermolecular bonding, resulting in a lower inherent dissolution rate than the PRZ-FLA binary cocrystal in the buffers of pH 1.2, 4.0, and 6.8, which could decline the initial concentration of PRZ-FLA [134].

APIs form cocrystals with coformers through hydrogen bonds, which could disrupt the original hydrogen bonding between API molecules. Because of the higher free energy, greater molecular mobility and weaker intermolecular interaction, cocrystals often possess high solubilities and faster dissolution rates [51]. The solubilities of the mixed cocrystal curcumin–lysine of type a and b were higher than that of curcumin. The in vitro dissolution results showed that the curcumin cocrystal powder dissolved rapidly in the pH 6.8 phosphate buffer. More than 95% of curcumin could be released from the mixed cocrystal, while the pure curcumin powder only dissolved about 40% in the dissolution buffer after five minutes [116].

### 6.2. Hygroscopicity

**Figure 12:**
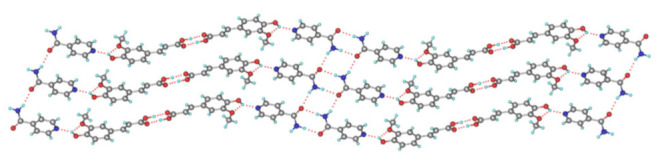
Crystal packing of FA-INA cocrystal showing hydrogen-bonded sheet structure mediated by acid–acid and amide–amide homosynthons and hydroxyl-pyridine heterosynthon [100] (With permission from [100]; Copyright 2020 Elsevier).

Aitipamula et al. prepared ferulic acid–isonicotinamide (FA-INA) cocrystal to lower hygroscopicity [100]. At 40 °C/75% RH, the stability of FA-INA cocrystal after three months of storage was significantly better than that of FA-INA physical mixture. The results showed that the moisture absorption rate of FA-INA cocrystal was less than 0.2% and did not convert to hydrates, indicating that the cocrystal is stable. In the asymmetric unit of the cocrystal structure, FA and INA molecules formed supramolecular amide–amide and acid–acid homozygotes, respectively. The amide–amide synthon further dimerized along the crystalline α axis and forms a chain of tetrameric units composed of INA molecules. The hydroxyl group of FA formed an O–H⋯N hydrogen bond with the pyridine N of INA and connected INA molecules to form a hydrogen bond chain (Figure 12). It was illustrated that formation of the cocrystal hydrate requires breaking of their original hydrogen bonds in anhydrous cocrystal and formation of strong hydrogen bonds, which was not possible within the time scale of a DVS experiment.

Wong et al. prepared curcumin–resorcinol and curcumin–pyrogallol cocrystals, both of which showed stability against high RH (no deliquescent under 95% relative humidity), while curcumin would obviously absorb moisture and increase weight under RH 75% [93]. The reduction of the hygroscopicity can be explained by the interaction between the phenol groups of coformers (resorcinol and pyrogallol) and the phenol groups and carbonyl groups of curcumins to form phenol–phenol intermolecular hydrogen bonds and phenol–carbonyl intermolecular hydrogen bonds. This makes it hard for the interaction between the phenolic groups in curcumin and water vapor molecules through O–H⋯O hydrogen bonds. Therefore, a stronger crystal lattice contributes to better moisture resistance.

**Figure 13:**
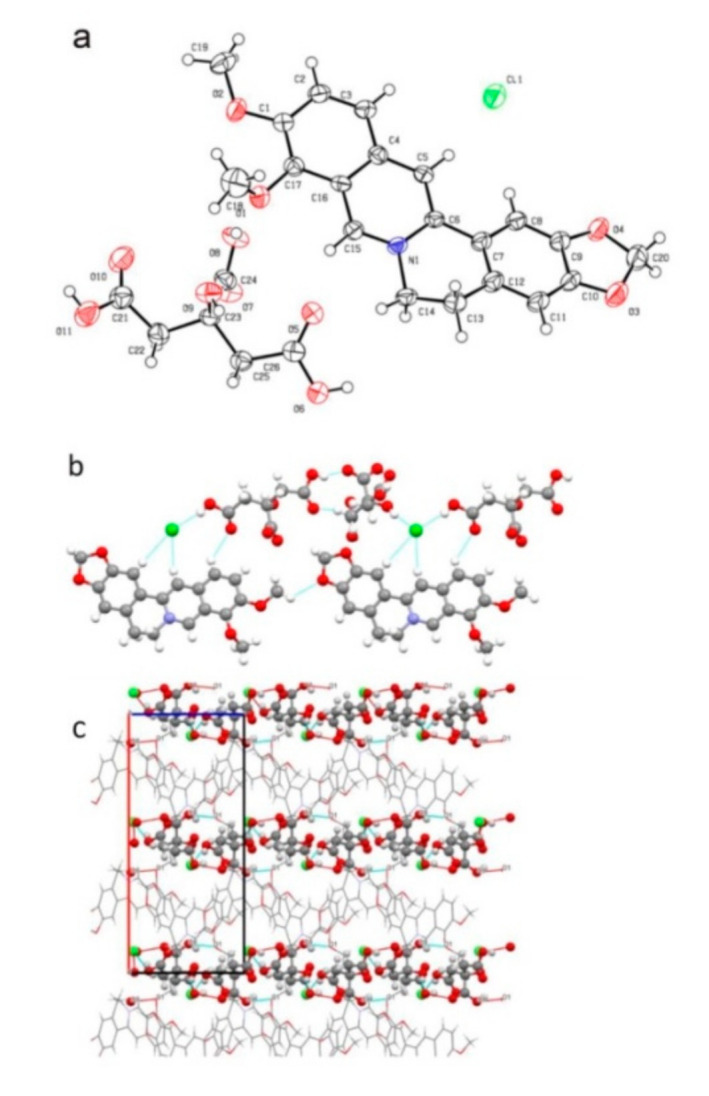
The crystal structures of BCl–CA cocrystal: (**a**) thermal ellipsoid drawing including atomic labelling scheme, (**b**) key intermolecular interactions (hydrogen bonds are teal colored), (**c**) molecular packing [60] (with permission from [60]; Copyright 2019 Elsevier).

Berberine chloride–citric acid (BCl-CA) cocrystal was advantageous over BCl·2H_2_O in term of physical stability against variations in RH. The crystal packing of BCl-CA cocrystal was stabilized by a dense hydrogen bond network consisting of O–H⋯Cl^–^ and O–H⋯O intermolecular hydrogen bonds, thereby fixing berberine chloride on the CA layer. Therefore, the crystal structure of BCl-CA consisted of alternating layers of CA and BCl [60]. Each chloride ion in BCl-CA interacted with two carboxylic acid groups in adjacent CA molecules (Figure 13). These interactions must be overcome if BCl-CA cocrystal wants to bind to water molecules. A higher energy barrier can prevent BCl-CA cocrystal from moisture sorption and hydrate formation.

**Figure 14:**
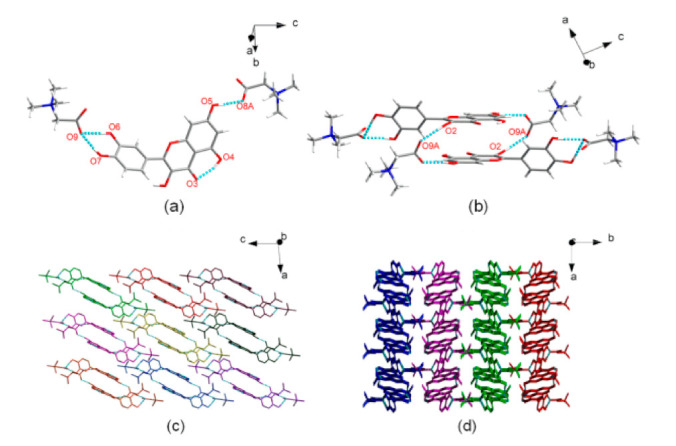
Crystal structure of QUE-BTN. (**a**) The asymmetric unit. (**b**) Two asymmetric units formed R^4^_4_ (24) dimeric structure. (**c**) 2D layered structure in the ac plane. Each dimeric structure in c is displayed in a different color. (**d**) 3D packing structure viewed along the *c* axis. In (**d**), adjacent molecular layers are displayed in different colors [71] (with permission from [71]; Copyright 2019 American Chemical Society).

Zhang et al. made quercetin–betaine cocrystal [71], and two nonequivalent betaine (BTN) molecules formed O7–H7⋯O9, O6–H6⋯O9, O2–H2⋯O9A, and O5–H5⋯O8A interactions. This mode of interaction also resulted in a short intramolecular (O6) H6⋯H7 (O7) distance (1.94). Two asymmetric units formed an R^4^_4_ (24) dimeric structure through two O2–H2⋯O9A hydrogen bonds (Figure 14b). The dimeric units were arranged in a parallel and staggered from head to tail, composing the layered structure in the ac-plane (Figure 14c). The layered and inverted structure was further packed alternately along the b axis to form a 3D arrangement of the crystal structure (Figure 14d). QUE-BTN could maintain its crystal phase when the RH was lower than 75%. It is believed that this hydrogen bonding and molecular packing of the cocrystal can prevent the combination of quercetin and external water molecules to a certain extent. Compared with the pure form of flavonoids, all cocrystals appeared to exhibit improved solubilities.

## 7. Conclusions and Future Perspectives

The physicochemical properties of T-APIs, such as solubility, dissolution, stability and bioavailability, play an important role in their safety and efficacy. Cocrystallization is a promising formulation strategy for resurrection of T-APIs with high therapeutic potentials but suffering from poor pharmaceutical properties. The majority groups of T-APIs used in cocrystal engineering involve flavonoids, followed by phenolic acids, and relatively few alkaloids and terpenoids, providing abundant hydrogen bond donors and acceptors for cocrystal formation. In addition, flexible dosing regimens are possible for T-APIs as their dosing control are less strict when compared to drugs, allowing more flexibility in T-API cocrystallization. At present, most studies about T-API cocrystals are still in an early preclinical stage, focusing on screening, solid state characterization, and simple pharmaceutical evaluation. However, the mechanism guiding the TCM cocrystal design, novel TCM cocrystal formulations (e.g., modified release and inhalable TCM cocrystals), and comprehensive in vitro and in vivo evaluation remains to be largely unexplored.

In addition, the long-term clinical experience of TCMs for thousands of years has shown that they have a good biocompatibility with the human body. Many T-APIs could be designed for combination therapy in the form of drug–drug or herbal–drug cocrystals to exert potentially synergistic therapeutic effects along with improving manufacturability and stability. Moreover, the abundant T-API compounds could serve as a useful database for virtual screening as many T-APIs are structurally similar.

In conclusion, the present review provides up-to-date information regarding the pharmaceutical applications of T-API cocrystals and mechanisms for modulating physicochemical properties through cocrystallization. With continuous research effort in expediting the basic theory and applications of cocrystals, it is believed that increasingly more innovative T-API cocrystals could be developed as a better treatment option for various diseases.

## Figures and Tables

**Table 1:** The problematic physiochemical properties of some T-APIs as new potential drugs [9].

Name of T-APIs	Main Sources of Plants	Problematic Physicochemical Properties	Major Indications
**Alkaloids**			
**Dauricine**	*Menispermum dauricum* DC.	Slightly soluble	Tachyarrhythmia
**Lycorine**	*Lycoris radiata* (L’Her.) Herb., *Narcissus tazetta* Linn. var. *chinensis* M.Roener, *Zephyranthes candida* Herb., *Crinum asiaticum* Linn. var. sinicum (Roxb.ex Herb.) Baker, *Galanthus woronawii* Losink., *Clivia miniata* Regel.	Practically insoluble	Intestinal and external amoeba
**Matrine**	*Sophora flavescens* Alt., *Euchresta japonica* Hook. f. ex Regel.	-	Chronic cervicitis, dysentery, enteritis, skin disease
**Sophocarpine**	*Sophora alopecuroides* Linn., *Sophora flavescens* Alt., *Sophora japonica* L., *Sophora davidii* (Franch.) Skeels.	Slightly soluble	Cancer, chronic bronchial asthma, malignant mole
**Toddaline**	*Chelidonium majus* Linn., *Toddalia asiatica* (L.) Lam.	Slightly soluble	Rheumatic pain
**Flavonoids**			
**Apigenin**	*Apium graveolens* Linn., *Veronica linariifolia* Pall.ex Link subsp.*dilatata* (NakaietKitag.) Hong., *Reynoutria japonica* Houtt., *Veratrum grandiflorum* Loes.	Practically insoluble	HIV and other viral infections, inflammation
**Baicalein**	*Scutellaria baicalensis* Georgi, *Oroxylum indicum* (Linn.) Bentham ex Kurz, *Plantago major* Linn.	Practically insoluble, unstable, prone to oxidation	Fever, sore throat, and upper respiratory tract infection
**Chrysin**	*Oroxylum indicum* (Linn.) Bentham ex Kurz, *Pinus mon-ticola* Dougl., *P.aristata* Engelm.	Practically insoluble	Cardiovascular and cerebrovascular diseases, inflammation
**Dihydromyricetin**	*Ampelopsis grossedentata* (Hand-Mazz)WT Wan	Hygroscopic, prone to oxidation and decomposition	Alcoholism, alcoholic liver, fatty liver
**Genistein**	*Euchresta japonica* Hook. f. ex Regel, *Genista tinctoria* Linn.	Practically insoluble	Cancer
**Hesperidin**	*Citrus sinensis* (Linn.) Osbeck, *Citrus limon* (L.) Burm. F.	Practically insoluble	Various diseases related to venous and lymphatic insufficiency, hypertension, and myocardial infarction
**Kaempferol**	*Kaempferia galanga* Linn.	Slightly soluble	Osteoclast bone resorption
**Luteolin**	*Reseda odorata* Linn., *Lonicera japonica* Thunb., *Dendranthema morifolium* (Ramat.) Tzvel., *Nepeta cataria* Linn., *Ajuga nipponensis* Makino.	Slightly soluble, low bioavailability	Cardiovascular disease, amyotrophic lateral sclerosis
**Myricetin**	Myricaceae, Vitaceae, Leguminosae, Ericaceae, and Euphorbiaceae.	Slightly soluble, low bioavailability	Cardiovascular disease
**Naringenin**	*Amacardi-um occidentale* L., *Prunus yedoensis* Mats., *Armeniaca mume* Sieb.	Practically insoluble, low bioavailability	Bacterial infection, cough, cancer
**Quercetin**	*Sophora japonica* Linn., *Platycladus orientalis* (Linn.) Franco, *Alpinia officinarum* Hance, *Tussilago farfara* Linn., *Taxillus sutchuenensis* (Lecomte) Danser, *Ginkgo biloba* Linn., *Sambucus williamsii* Hance, etc.	Practically insoluble, low bioavailability	Bacterial infection, viral infection, tumor, diabetes, hyperlipidemia, and immune system diseases
**Phenolic acids**			
**Curcumin**	*Curcuma aromatica* Salisb., *Curcuma longa* Linn., *Curcuma zedoaria* (Christm.) Rosc., *Acorus calamus* Linn.	Practically insoluble, poor stability, prone to degradation, poor solubility in acidic condition, low dissolution rate and bioavailability	Inflammatory bowel disease, pancreatitis, arthritis
**Ferulic acid**	*Ferula assafoetida* L., *Angelica sinensis* (Oliv.) Diels, *Ligusticum chuanxiong* Hort., *Cimicifuga foetida* L., *Ziziphus jujuba* Mill. var. *spinosa* (Bunge) Hu ex H.F.Chow.	Practically insoluble	Cardiovascular diseases, cerebrovascular diseases, leukopenia, and other diseases
**Gallic acid monohydrate**	*Rhus chinensis* Mill.	Slightly soluble	High blood pressure, hyperlipidemia, colon cancer, skin tumors
**Oleanolic acid**	*Olea europaea* Linn., *Swertia mileensis* T. N. Ho et W. L. Shi, *Fructus Ligustri* Lucidi, *Hemsleya macrosperma* C. Y. Wu ex C. Y. Wu et C. L. Chen.	Practically insoluble	Bronchitis, pneumonia, acute tonsillitis, periodontitis, bacillary dysentery, acute gastroenteritis, urinary infection, acute hepatitis
**Pterostilbene**	*Pterocarpus indicus* willd., *Vitis vinifera* Linn., and *Ormosia henryi* Prain.	Practically insoluble	High blood pressure, hyperlipidemia, colon cancer, skin tumors
**Resveratrol**	*Reynoutria japonica* Houtt., *Cassia tora* Linn., *Morus alba* L.	Practically insoluble, photosensitive, thermally unstable	Cancer, high blood cholesterol
**Rhein**	Rheum officinale Baill.	Practically insoluble	Hyperlipidemia, constipation
**Ursolic acid**	*Prunella vulgaris* Linn., *Ilex rotunda* Thunb.	Practically insoluble	Viral hepatitis, depression, primary hyperlipidemia
**Salvianolic acid B**	*Salvia miltiorrhiza* Bge.	Photosensitive, thermally unstable, prone to degradation	Ischemic stroke
**Terpenoids**			
**Loganin**	*Strychnos nux-vomica* Linn.*, Cornus officinalis* Sieb. Et Zucc.	Practically insoluble	Cancer
**Triptolide**	*Tripterygium wilfordii* Hook. F.	Practically insoluble	Rheumatoid arthritis

Notes: According to the *British Pharmacopoeia*, <0.1 mg/mL refers to practically insoluble, 1–10 mg/mL refers to slightly soluble, both of which will lead to low bioavailability.

**Table 2:** Sources of plants, physicochemical problems, and major indications of T-APIs, including alkaloids, flavonoids, phenolic acids and terpenoids, recorded in Volume II, *Chinese Pharmacopoeia* (2020 edition) [36].

Name of T-APIs	Main Sources of Plants	Problematic Physicochemical Properties	Major Indications
**Alkaloids**			
**Berberine chloride**	*Coptis chinensis* Franch.*, Phellodendron amurense* Rupr.	Slightly soluble, low bioavailability, bitter taste	Diabetes, high blood pressure
**Bulleyaconitine A**	The subgenus Subgen. Aconitum.	Practically insoluble	Rheumatoid arthritis
**Homoharringtonine**	The genus Cephalotaxus.	Slightly soluble	Acute myelogenous leukemia, acute monocytic leukemia, malignant lymphoma
**Huperzine-A**	The genus Huperzia, i.e., *Huperzia serrata* (Thunb. ex Murray) Trev.	Practically insoluble, hygroscopic	Senile dementia, myasthenia gravis
**Pseudoephedrine hydrochloride**	*Ephedra sinica* Stapf.	-	Nasal congestion caused by cold, allergic rhinitis, rhinitis, sinusitis
**Raceanisodamine**	*Atropa belladonna Linn., Datura metel* L.	-	Choline drug resistance, smooth muscle spasm, gastrointestinal colic, biliary tract spasm, organophosphorus poisoning
**Reserpine**	The genus Rauvolfia.	Slightly soluble, photosensitive	Mild and moderate hypertension
**Tetrahydropalmatine sulfate**	*Corydalis yanhusuo* W. T. Wang.	Slightly soluble, prone to oxidation, photosensitive	medical ailments, prenatal labor pains, postpartum contractions, menstrual pain, headache, insomnia
**Ligustrazine hydrochloride**	*Ligusticum chuanxiong* Hort., *Curcuma aromatica* Salisb.	Prone to sublimation, hygroscopic	Vasodilator, occlusive cerebrovascul- ar disease, ischemic vascular disease
**Vinblastine sulfate**	*Catharanthus roseus* (Linn.) G. Don.	Hygroscopic, photosensitive, prone to thermal degradation	Hodgkin’s disease, chorioepithelial carcinoma, lymphoid sarcoma, acute leukemia, breast cancer, testicular tumor, choriocarcinoma
**Vincristine sulfate**	*Catharanthus roseus* (Linn.) G. Don.	Hygroscopic, photosensitive, prone to thermal degradation	Acute leukemia

**Flavonoids**			
**Baicalin**	*Scutellaria baicalensis* Georgi.	Slightly soluble, low bioavailability, poor tabletability	Acute and chronic hepatitis, persistent hepatitis, nephritis, pyelonephritis, and allergic diseases
**Rutinum**	*Sophora japonica* Linn., *Platycladus orientalis* (Linn.) Franco, *Alpinia officinarum* Hance, *Tussilago farfara* Linn., *Taxillus sutchuenensis* (Lecomte) Danser, *Ginkgo biloba* Linn., *Sambucus williamsii* Hance, etc.	Practically insoluble, hygroscopic	Hypertensive encephalopathy, cerebral hemorrhage, retinal hemorrhage
**Phenolic acids**			
**7-Hydroxy-4-methylcoumarin**	-	Practically insoluble	Cholecystitis, cholelithiasis, biliary tract infection, cholecystectomy syndrome
**Propyl gallate**	*Rhus chinensis* Mill.	Slightly soluble	Cerebral thrombosis, coronary heart disease, thrombophlebitis
**Terpenoids**			
**Andrographolide**	*Andrographis paniculata* (Burm. f.) Nees.	Practically insoluble	Upper respiratory tract infection, bacterial dysentery, bacterial and viral upper respiratory tract infections, dysentery
**Artemether**	*Artemisia annua* Linn.	Practically insoluble	Plasmodium falciparum, dangerous malaria resistant to chloroquine
**Artemisinin**	*Artemisia annua* Linn.	Practically insoluble, low bioavailability, short biological half-life, frequent drug administration is needed	Malaria, pulmonary hypertension
**Artesunate**	*Artemisia annua* Linn.	Slightly soluble	Cerebral malaria and various critical malaria
**Dihydroartemisinin**	-	Slightly soluble	Malaria Plasmodium falciparum, dangerous malaria resistant to chloroquine and piperaquine
**(R)-Camphor**	*Cinnamomum camphora* (Linn.) Presl.	Low aqueous soluable, volatile	Skin irritant
**Taxol**	Some various plants of the genus Taxus Linn.	Practically insoluble	Ovarian cancer, breast cancer, non-small cell lung cancer

Notes: According to the *British Pharmacopoeia*, <0.1 mg/mL refers to practically insoluble, 1–10 mg/mL refers to slightly soluble, both of which will lead to low bioavailability.

**Table 3:** Supramolecular interactions formed in the reported T-APIs.

Drug	Coformer	Single Crystal Structure	Supramolecular Interactions
**Alkaloids**			
**Berberine chloride**	Citric acid [60] Ibuprofen [61] Fumaric acid [62] Lactic acid * [63] Myricetin [64] Dihydromyricetin [64]	Reported - Reported - Reported Reported	−COOH···Cl - O−H···Cl, π–π interaction - O−H···Cl O−H···Cl
**Berberine**	Phthalic acid * [65] Chrysin [66]	- Reported	- O−H···O, interaction
**Ligustrazine**	Bexarotene [67]	Reported	O−H···N
**Flavonoids**			
**Apigenin**	4,4′-Bipyridine * [68]	Reported	O−H···N, π–π interaction
**Baicalein**	Nicotinamide [69] Caffeine [69] Isoniazid [69] Isonicotinamide [70] Theophylline [70] Betaine [71]	Reported Reported - Reported Reported Reported	O−H···N, O−H···O, N−H···O O−H···N, O−H···O, π–π interaction - O−H···N_arom_ O−H···N_arom_ O−H···O
**Chrysin**	Cytosine [72] Thiamine hydrochloride * [72]	Reported Reported	N−H···N N−H···O
**Dihydromyricetin**	Pentoxifylline [73] Caffeine [74] Urea* [74]	Reported - -	O−H···O - -
**Fisetin**	Nicotinamide [75] Isonicotinamide [75]	Reported Reported	O−H···N_arom_ O−H···N_arom_
**Genistein**	Isonicotinamide [76] 4,4′-Bipyridine * [77] Caffeine [78] Nicotinamide [75]	Reported Reported Reported Reported	O−H···N O−H···N O−H···N, O−H···O O−H···N_arom_
**Hesperidin**	Caffeine [79] Nicotinamide [79] Picolinic acid * [79] Temozolomide [80]	Reported Reported Reported Reported	O−H···N N−H···N_arom_, C=O···OH− C=O···OH− O−H···N
**Kaempferol**	5-Fluorouracil [81] 4,4′-Bipyridine * [81] Propylthiouracil [82]	Reported Reported Reported	O−H···N, O−H···O, N−H···O, C−H···O O−H···N, π–π interaction C−H···O
**Luteolin**	Isonicotinamide [75]	Reported	O−H···N_arom_
**Myricetin**	4,4′-Bipyridine * [81]	Reported	O−H···N, π–π interaction
**Naringenin**	4-Hydroxypyridine [83] Anthranilamide [83] Flavone [83] 4,4′-Bipyridine * [83] Isonicotinamide [84] Picolinic acid * [84] Betaine [84] Carbamazepine [85]	Reported Reported Reported Reported Reported - Reported Reported	O−H···C=O O−H···C=O O−H···C=O O−H···C=O O−H···N, N−H···O - O−H···O O−H···O, N−H···O
**Puerarin**	Lornoxicam [86]	-	-
**Quercetin**	4,4′-Bipyridine * [81] Caffeine [7] Isonicotinamide [7] Theobromine dihydrate [7] Isoniazid [87] Nicotinamide [88] Pyrazole * [89] Imidazolidinone * [89] Baclofen [89]	Reported Reported Reported - Reported - - - -	O−H···N, π–π interaction O−H···O, O−H···N_arom_ O−H···C=O, O−H···N_arom_ - N−H···O - - - -
**Phenolic acids**			
**Curcumin**	Isoniazid [90] Hydroquinone * [91] Phloroglucinol [92] Resorcinol [93] Pyrogallol [93] Ascorbic acid [94] Trimesic acid [95]	- - - Reported Reported - -	- - - O−H···O, C−H···O, C−H···π O−H···O, C−H···O, C−H···π - -
**Gallic acid**	Metronidazole [96] Lenalidomide [97] Isoniazid [98]	Reported Reported Reported	O−H···O O−H···O, N−H···O O−H···O, O−H···N, π–π interaction
**Ferulic acid**	Pyrazinamide [99] Urea [100] Nicotinamide [100] Isonicotinamide [100]	Reported Reported Reported Reported	C=O···N−H N−H···N O−H···NO−H···N
**Oleanolic acid**	Isoniazid [101]	-	-
**Pterostilbene**	Ethylenediamine * [102] Caffeine [103] Carbamazepine [103]	Reported Reported Reported	C−H···π O−H···N, O−H···O O−H···N, O−H···O
**Resveratrol**	Curcumin [104] Isoniazid [105] 4-Aminobenzamide * [106]	- Reported -	- N−H···O -
**Ursolic acid**	Ethylenediamine * [107] Piperazine [108] Metformin [109] Arginine [109] Lysine [109] N-Methylglucamine [109]	- - - - - -	- - - - - -
**Terpenoids**			
**Andrographolide**	Salicylic acid [110] Vanillin [110] Vanillic acid [110] Guaiacol [110] Resorcinol [110]	Reported Reported Reported Reported Reported	O−H···O, C−H···O O−H···O, C−H···O O−H···O, C−H···O O−H···O O−H···O
**Artemisinin**	Orcinol [18] Resorcinol [18]	Reported -	C−H···O, O−H···π C−H···O, O−H···O
**Artesunate**	Nicotinamide [111]	-	-
**11-Aza-artemisinin**	Benzoic acid [54] Salicylic acid [54] Succinic acid [54] Heptanedioic acid [54]	- - - -	- - - -

Note: * indicates belonging to non-GARS or non-drug compounds.

## Abbreviations

11-AZA, 11-azartemisinin; ACS, acesulfame; AP, andrographolide; APIs, active pharmaceutical ingredients; ART, artemisinin; BbAs, berberine acesulfame; BB, berberine; BbSac, berberine saccharine; BCD, BCL dihydrate; BCl, berberine chloride; BCS, biopharmaceutics classification system; BCT, BCl tetrahydrate; BEN, benzoic acid; BER, berberine chloride; Beta-Chlor^®^, chloral betaine; BTN, betaine; CA, citric acid; CAF, caffeine; Cafcit^®^, caffeine citrate; CAF·MeOH, caffeine·MeOH; CSD, Cambridge Structural Database; CP, *Chinese Pharmacopoeia*; CUR, curcumin; Depakote^®^, valproate semisodium; DMY, dihydromyricetin; DSC, differential scanning calorimetry; FA, fumaric acid; FDA, Food and Drug Administration; FLA, ferulate; GRAS, generally recognized as safe; HQ, hydroquinone; IDR, intrinsic dissolution rate; INA, isonicotinamide; INH, isoniazid; ISN, isoniazid; Lexapro^®^, escitalopram oxalate; MYR, myricetin; NAM, nicotinamide; NCT, nicotinamide; ORC, orcinol; PHBA, p-hydroxybenzoic acid; PHO, phosphate; PRA, pyrazinamide; PRZ, piperazine; PXRD, powder X-ray diffraction; QUE, quercetin; RES, resorcinol; SAC, saccharine; SLA, salicylic acid; TA, L-tartaric acid; T-APIs, active pharmaceutical ingredients of traditional Chinese medicine; TCMs, traditional Chinese medicines; TGA, thermal gravimetric analysis; TMP, ligustrazine (tetramethylpyrazine).
